# Biofilm Eradication Using Biogenic Silver Nanoparticles

**DOI:** 10.3390/molecules25092023

**Published:** 2020-04-26

**Authors:** María Belén Estevez, Sofía Raffaelli, Scott G. Mitchell, Ricardo Faccio, Silvana Alborés

**Affiliations:** 1 Área de Microbiología, Departamento de Biociencias, Facultad de Química, Universidad de la República, Montevideo 11800, Uruguay; 2 Instituto de Ciencia de Materiales de Aragón (ICMA), Consejo Superior de Investigaciones Científicas (CSIC)-Universidad de Zaragoza, 50009 Zaragoza, Spain; 3 CIBER-BBN, Instituto de Salud Carlos III, 28029 Madrid, Spain; 4 Centro NanoMat & Grupo Física, Departamento de Experimentación y Teoría de la Estructura de la Materia y sus Aplicaciones (DETEMA), Facultad de Química, Universidad de la República, Montevideo 11800, Uruguay

**Keywords:** biogenic nanoparticles, silver nanoparticles, antimicrobial, antibiofilm

## Abstract

Microorganisms offer an alternative green and scalable technology for the synthesis of value added products. Fungi secrete high quantities of bioactive substances, which play dual-functional roles as both reducing and stabilizing agents in the synthesis of colloidal metal nanoparticles such as silver nanoparticles, which display potent antimicrobial properties that can be harnessed for a number of industrial applications. The aim of this work was the production of silver nanoparticles using the extracellular cell free extracts of *Phanerochaete chrysosporium*, and to evaluate their activity as antimicrobial and antibiofilm agents. The 45–nm diameter silver nanoparticles synthesized using this methodology possessed a high negative surface charge close to −30 mV and showed colloidal stability from pH 3–9 and under conditions of high ionic strength (*[NaCl]* = 10–500 mM). A combination of environmental SEM, TEM, and confocal Raman microscopy was used to study the nanoparticle-*E. coli* interactions to gain a first insight into their antimicrobial mechanisms. Raman data demonstrate a significant decrease in the fatty acid content of *E. coli* cells, which suggests a loss of the cell membrane integrity after exposure to the PchNPs, which is also commensurate with ESEM and TEM images. Additionally, these biogenic PchNPs displayed biofilm disruption activity for the eradication of *E. coli* and *C. albicans* biofilms.

## 1. Introduction

Biological synthesis is an alternative green technology used for the production of metal nanoparticles, compared to the conventional physical and chemical methods that may use toxic and/or expensive reagents and severe reaction conditions [1]. Some microorganisms are able to accumulate and detoxify heavy metals due to various reductase enzymes, which reduce metal salts to metal nanoparticles with a narrow size distribution. For this reason, microorganisms have immense potential as ecofriendly and cost-effective tools for biological synthesis of nanoparticles, avoiding toxic, harsh chemicals and the high energy demand required for physiochemical synthesis [2] Biological synthesis by microorganisms includes the intracellular or extracellular production [3,4]. Extracellular synthesis of nanoparticles has received much attention because it eliminates the downstream processing steps required for the recovery of nanoparticles in intracellular methodologies, such as sonication to break down the cell wall and several centrifugation and washing steps required for nanoparticle purification. Additionally, extracellular peptides, enzymes, reducing cofactors, and organic materials have significant roles by acting as reducing agents, providing natural capping to synthesize nanoparticles [5]. Crucially, such capping prevents nanoparticle aggregation and helps them to remain stable for long periods of time [2].

The commercial interest in silver nanoparticles lies in their various applications in diverse fields where their potent antimicrobial properties can be harnessed [6]. Recently there has been a drive towards the synthesis of silver nanoparticles by microorganisms [7,8]. In a previous study, our group reported an extracellular cell-free filtrate method for the biological synthesis of silver nanoparticles. The fungal strains were selected based on their silver nanoparticle synthesis capacity, yield of production, resistance to centrifugation and long-term colloidal stability of the obtained nanoparticles following their purification protocols. Moreover, these nanoparticles showed antibacterial activity against *Escherichia coli* in agar diffusion assays [7].

Another important aspect of the antimicrobial properties of silver nanoparticles is their potential to eradicate or inhibit microbial biofilm formation, which is an important virulence factor in many localized chronic infections [9,10]. One of the most notable emergent biofilm properties is that adhering microorganisms can embed themselves in a self-produced matrix of extracellular polymeric substances (EPS) composed of proteins, polysaccharides, humic acids and DNA. This matrix of EPS acts as the glue that holds together bacterial biofilms, protecting them against host immune systems and environmental challenges, among other exogenous threat to survival and proliferation. Consequently, the polymeric matrix plays a crucial role in antimicrobial resistance by preventing antibiotic penetration inside the biofilm. Despite the EPS-matrix containing water-filled channels required for the exchange of nutrients and metabolic waste-products with their environment, most antimicrobials have difficulty penetrating EPS, either through size limitations or ready adsorption onto the EPS-matrix or to bacterial cell surfaces that can be highly negatively charged at physiological pH, but becoming more positively charged with decreasing pH inside a biofilm. Such phenomena help the survival of internal biofilm cells and illustrate the limitation of traditional molecular approaches to biofilm eradication. In this sense, advances in nanotechnology can provide a great tool [11,12]. For example, reports on silver nanoparticles have shown they are able to diffuse toward the inner part of the biofilm before releasing Ag^+^ ions, where this penetration leading to a high kill rate against the bacteria in the lower layers of the biofilm [13].

Different nanomaterials, non-adhesive, drug-releasing and contact-killing coatings, are under design to prevent biomaterial-associated infection of implants such as artificial hips and knees. Moreover, nanotechnology is looked at as an extremely promising way to create new antimicrobials and delivery systems, able to penetrate biofilms and kill multidrug resistant strains [14]. Size, shape and surface properties of the resulting nanomaterials are important to consider with respect to the control of biofilm-infection [15]. Nanoparticle size is important for penetration into biofilms [13]; the ideal diameter for NPs in biofilm-infection control would range between five and 100–200 nm, and not exceeding 500 nm [14].

Antimicrobial and antibiofilm mechanisms of silver nanoparticles are not completely known, and they depend on the type of microorganism and the physicochemical properties of the nanoparticle [16,17]. Nanoparticles are able to contact with bacterial cell walls through different interactions, cross microbe membranes, interfere with metabolic pathways, induce changes in membrane shape and function, interact with the microbial cellular machinery to inhibit enzymes, deactivate proteins, induce oxidative stress and electrolyte imbalance, or modify gene expression levels. Given their enormous therapeutic potential, understanding the modes of action responsible for the bactericidal properties of NPs becomes imperative [18]. Furthermore, it is also important to develop accessible, cheap, rapid and reliable methods as alternatives to traditional biochemical methods which often determine only one mechanism of action at sub-cellular level. For this purpose, we used three complementary techniques: transmission electron microscopy (TEM), environmental scanning electron microscopy (ESEM), and confocal Raman microscopy (CRM). Raman spectroscopy provides us with molecular fingerprint information and permits us to study subtle changes in cellular structure, as a result of modification of proteins, lipids and nucleic acids that undergo changes in their Raman band profiles. This technique has been used to provide an image of the antimicrobial effect and was recently used to study molecular changes within bacterial cells after antibiotic treatment [19,20].

The aim of this work was to produce silver nanoparticles using the extracellular cell free extracts of *Phanerochaete chrysosporium* and evaluate their antimicrobial and antibiofilm properties. To achieve this, our study focused on the interactions between the biogenic silver nanoparticles (PchNPs) and *E. coli* cells using TEM, ESEM and CRM, in order to ascertain the antimicrobial mechanisms of these nanoparticles; while the effect of these antimicrobial nanoparticles on the disruption and eradication of *E. coli* and *C. albicans* biofilms was also evaluated.

## 2. Results and Discussion

### 2.1. Synthesis of Biogenic Silver Nanoparticles (PchNPs)

**Figure 1:**
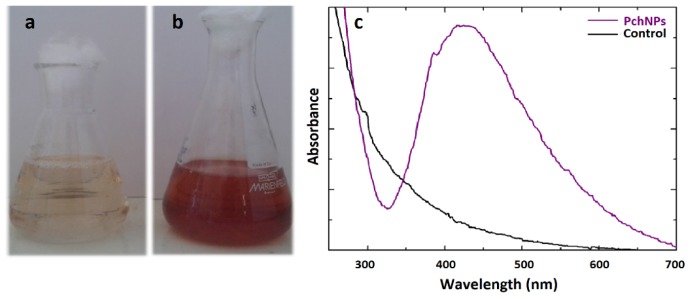
Color change obtained for silver nanoparticles synthesized (**a**) control, (**b**) PchNPs and (**c**) UV-Vis absorption spectra, after 24 h of reaction.

The biomass from cultures of the fungus *Phanerochaete chrysosporium* was harvested, washed and suspended in sterilized distilled water. After incubation, the cell-free filtrate was added to a silver nitrate solution and the mixture was incubated in dark. The absorbance spectrum was measured in the range of 250–800 nm and the maximum peak was determined. Color change in the reaction mixture (Figure 1a,b) as well as the appearance of an absorption band between 400 and 450 nm corresponding to the surface plasmon resonance (SPR) (Figure 1c) were indicative of the formation of silver nanoparticles (Figure 1b).

**Figure 2:**
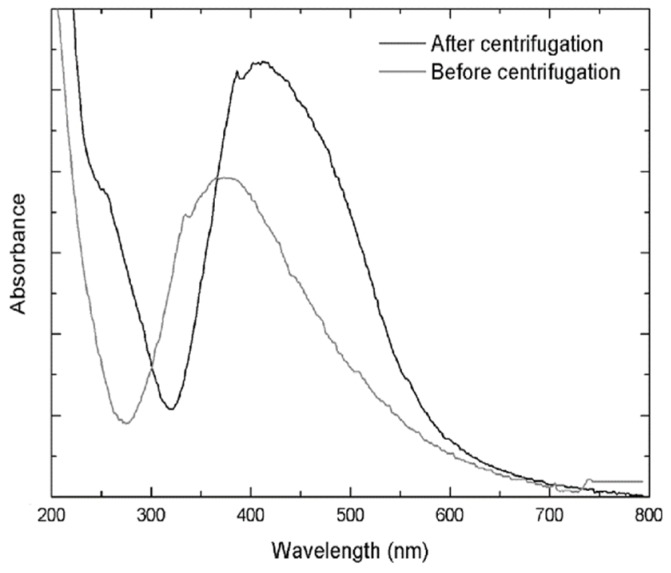
UV-visible absorption spectrum before and after centrifugation showing the final SPR band of the resuspended PchNPs at 442 nm, corresponding to the stable silver nanoparticles.

After a 24 h period an 87% yield was obtained from the synthesis. The reaction mixture was centrifuged and the pellet containing the silver nanoparticles was washed with and resuspended in water. The UV-vis spectra after centrifugation showed an absorption band at 442 nm, corresponding to the SPR band of silver nanoparticles (Figure 2).

### 2.2. Evaluation of the Incidence of Reaction Conditions in the Biosynthesis of PchNPs

**Figure 3:**
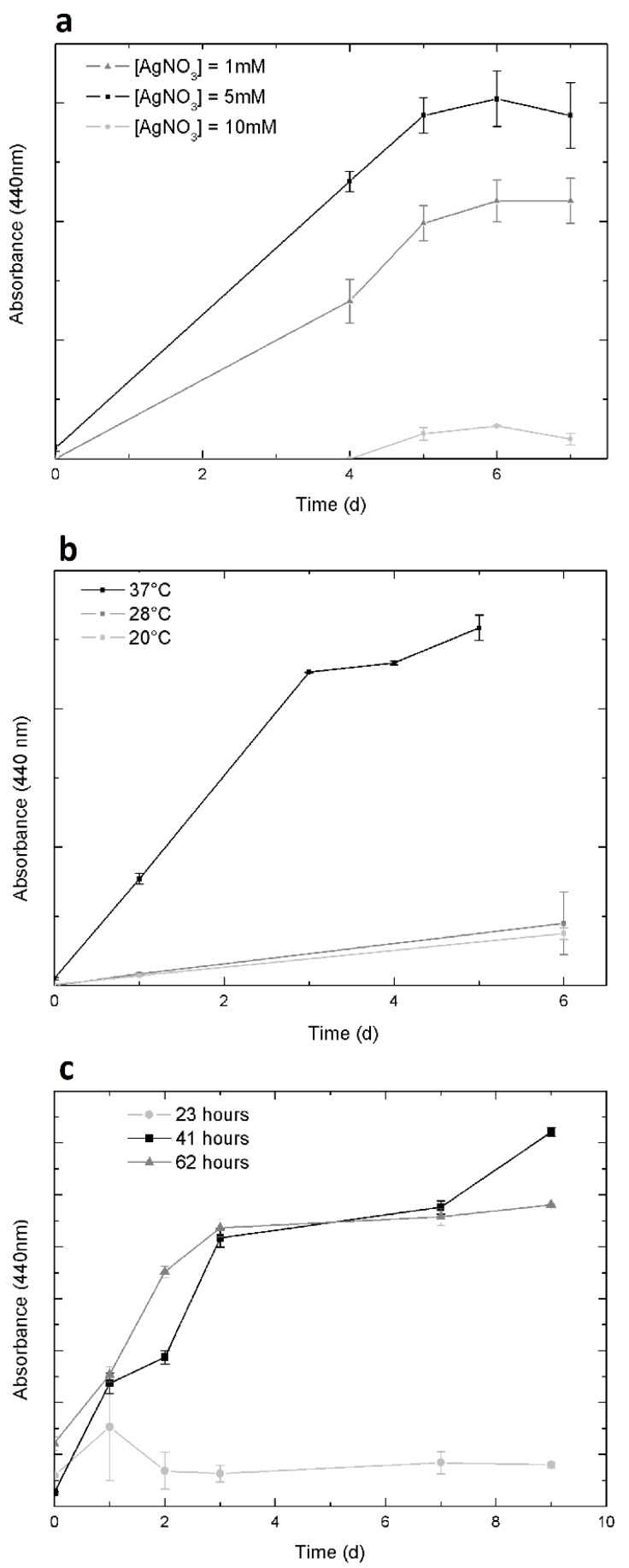
Effect of different reaction conditions on the synthesis of PchNPs (measured by absorbance at 440 nm) over several days, where error bars indicate the standard deviation: (**a**) concentration of AgNO_3_, (**b**) reaction temperatures, (**c**) incubation times of the fungal mycelium with water.

The evolution of silver nanoparticle synthesis over time was monitored by measuring the formation of SPR band via UV-vis spectroscopy absorbance at 440 nm. The incidence of AgNO_3_ concentration, reaction temperature and incubation time of the mycelium with water are shown in Figure 3. 5 mM AgNO_3_ was the concentration that reached the highest yield for the nanoparticle synthesis (Figure 3a). In addition, the conditions for the greatest nanoparticle production in the shortest time found at a reaction temperature of 37 °C (Figure 3b) and 41 h of incubation of the mycelium with water (Figure 3c). All subsequent biosyntheses were carried out using these conditions to obtain the highest production of stable PchNPs in the shortest time.

### 2.3. Characterization of PchNPs

#### 2.3.1. ζ-potential: Surface Charge of PchNPs

ζ-potential determination showed that PchNPs had a high net negative surface charge, close to −30 mV (−24.8 mV), indicating their colloidal stability in aqueous media.

#### 2.3.2. DLS and SAXS: Diameter of PchNPs

**Figure 4:**
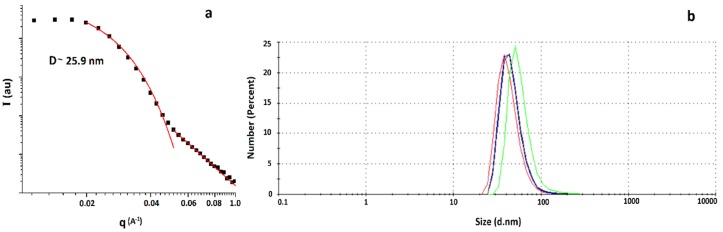
(**a**) SAXS curve showing one characteristic correlated distance and (**b**) DLS measurement indicating nanoparticles of ca. 45 nm diameter in solution with low polydispersity.

According to the Small Angle X-Ray Scattering (SAXS) measurement, silver nanoparticle size was 26(2) nm (Figure 4a). DLS characterization showed a single population with a hydrodynamic diameter of ca. 45 nm (Figure 4b), larger than the diameter obtained using SAXS, which is to be expected, since DLS measures the hydrodynamic diameter of the nanoparticles.

#### 2.3.3. Confocal Raman Microscopy: Surface Functional Groups of PchNPs

**Figure 5:**
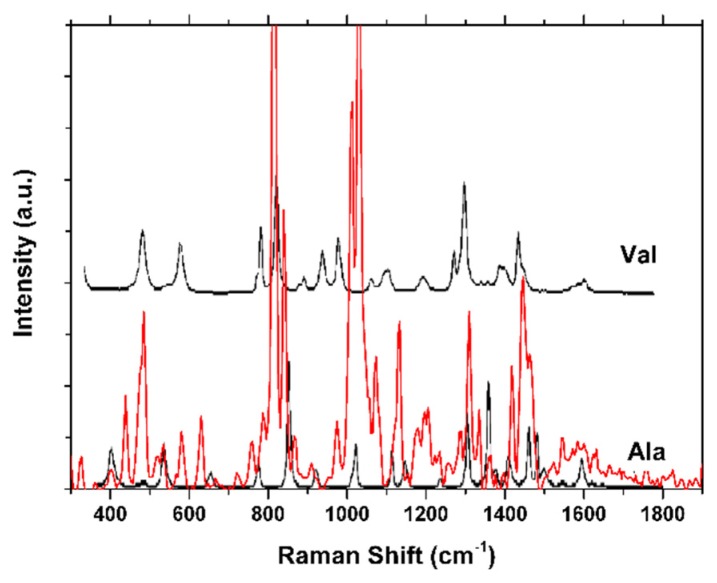
Raman spectrum for PchNPs with superimposed Raman spectra of l-valine and l-alanine.

The use of fungi for silver nanoparticle production provides improved production capacity due to the high quantities of secreted substances; these bioactive substances play a dual functional role as reducing and stabilizing agents to the colloidal nanoparticles [21]. Confocal Raman Microscopy was used to characterize the surface functional groups capping of the silver nanoparticles (Figure 5). In particular, the nature of silver nanoparticles gives rise to Surface-Enhanced Raman Scattering (SERS) phenomena, which produce a local field effect at the surface of the nanoparticle. Due to this amplifier effect, it is possible to enhance the Raman signal of those molecular fragments near the surface of the nanoparticles, which is of great importance to assess the nature of the stabilizing agent (capping) [22]. The presence of the band positioned at 230 cm^−1^ can be attributed to the Ag-N stretching mode, which may be originated from the fungal extract, providing the nitrogen atoms that will finally take part of the capping of the silver nanoparticle. Even more, bands positioned at the fingerprint region can be attributed to the presence of l-valine and l-alanine, which indicate that proteins from the fungal extract are part of the capping, or are near the nanoparticle metallic surface [22].

### 2.4. Colloidal Stability Assays

**Figure 6:**
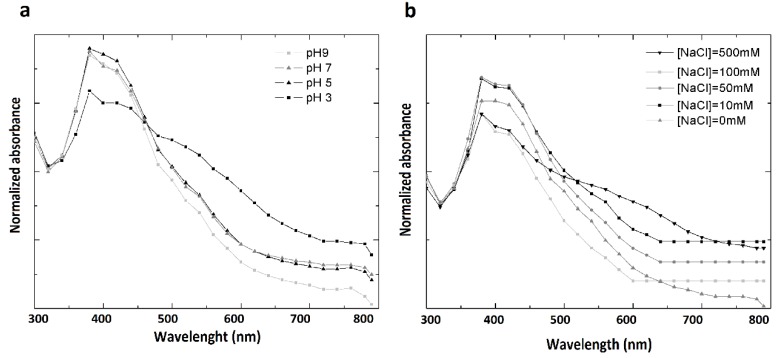
Stability of PchNPs (**a**) from pH 3 to 9, and (**b**) at different ionic strength, *[NaCl]* 0–500 nM

The colloidal stability of PchNPs over pH 3–9 and under conditions of high ionic strength (*[NaCl]* = 10–500 mM) were evaluated (Figure 6a,b respectively). Aggregation of the nanoparticles generates a decrease in intensity at the Surface Plasmon Resonance (SPR) band, as well as a shift in the visible spectrum towards the infrared. Although PchNPs were stable for most of the evaluated conditions, a decrease at the band corresponding to the SPR band, as well as a slight broadening of the peak, were observed at 100 and 500 mM NaCl and pH 3. These results could be attributed to the neutralization of surface charges of PchNPs, which presented negative net charge at pH 6, resulting in their aggregation.

### 2.5. Antimicrobial Activity of PchNPs against E. coli

#### 2.5.1. Antibacterial Activity against *E. coli*

In order to determine the antibacterial activity of PchNPs, a bacterial inoculum of *E. coli* in LB media was supplemented with different concentrations of PchNPs and incubated for 24 h. Consistent with results reported by other authors using biogenic nanoparticles [23], in this work the Resazurin cell viability assays showed minimum inhibitory concentration (MIC) of PchNP of 0.25 nM for *E coli* cells.

#### 2.5.2. ESEM and TEM of *E. coli* Cells Exposed to PchNPs

**Figure 7:**
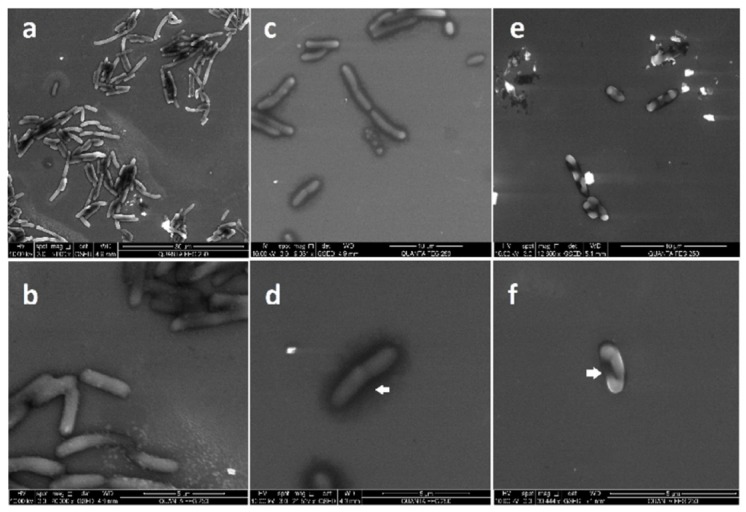
ESEM images of the interaction between *E. coli* and PchNPs. (**a**,**b**): control, (**c**–**f**): *E. coli* treated with PchNPs (**c**,**d**: 0.12 nM and **e**,**f**: 0.25 nM). Membrane puncturing and cell lysis indicated by white arrows. Note that panel **e** contains bright artifacts from the glutaraldehyde fixation.

**Figure 8:**
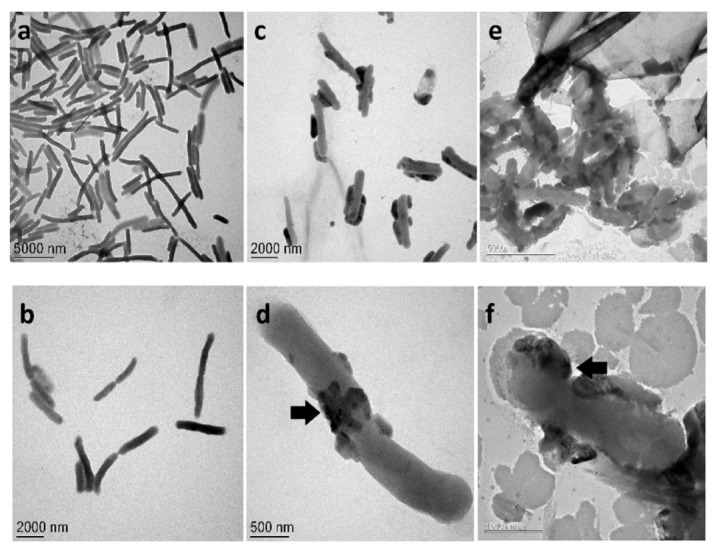
TEM images of the interaction between *E. coli* and PchNPs. (**a**,**b**): control, (**c**–**f**): *E. coli* treated with PchNPs (**c**,**d**: 0,12 nM and **e**,**f**: 0,25 nM). Black arrows indicate cell membrane puncturing. Panel **f** shows a significant amount of debris from cell lysis in addition to the bacterial cells.

Based on the results of antibacterial assays, we opted to use electron microscopy techniques (TEM and ESEM) to evaluate the interaction of the silver nanoparticles (at 0.12 and 0.25 nM) with *E. coli*. ESEM imaging shows that when *E. coli* cells are exposed to PchNPs (Figure 7c–f), their cell wall shows loss of integrity when compared to the control (Figure 7a,b). At lower, sub-MIC, PchNPs concentrations (Figure 7c,d), bacterial cells appear to be losing part of their cytoplasmic components (indicated by arrows in Figure 7d) and, with the increment of the concentration (Figure 7e,f), the damage of the cell membrane is clearly evidenced (indicated by arrows in Figure 7f). Meanwhile TEM images demonstrate that at the lowest PchNP concentration of 0.12 nM (Figure 8c,d), bacterial cells possess large conglomerates of silver nanoparticles on their surface (indicated by arrows) and show distinctive morphological changes when compared to control (Figure 8a,b).

Increasing silver nanoparticle concentration to 0,25 nM (Figure 8e,f) resulted in a decrease in the integrity of the cell membrane (indicated by black arrows in Figure 8d,f), probably as a consequence of the loss of cytoplasmic components due to degeneration of pores on the cell, as shown in the ESEM images.

### 2.6. Antimicrobial Properties of PchNPs Using Confocal Raman Microscopy

Studies of the phenotypic profile of *E. coli* and *C. albicans* cells before and after treatment with silver nanoparticles were carried out using confocal Raman microscopy. In order to evaluate the antimicrobial activity of PchNPs we selected two very different microorganisms (a prokaryotic and a eukaryotic microorganism). An important structural difference between them, that is the target of action of several antimicrobials, is the composition of the cell envelope. *E. coli* cells have a complex cell envelope composed of the cytoplasmic membrane, the periplasm with a thin peptidoglycan layer, and the outer membrane. In *C. albicans* the major components of the cell wall are fibrillar polysaccharides and proteins, with an inner layer enriched for chitin and polysaccharide matrix and outer layers enriched for mannoprotein. Minimum inhibitory concentration (MIC) of the nanoparticles was determined. MIC of PchNPs were 3.0 × 10^−2^ nM against *E. coli* and *C. albicans*. Both microorganisms tested were more sensitive to the PchNPs than to AgNO_3_ solution (MIC 7.8 µM).

**Figure 9:**
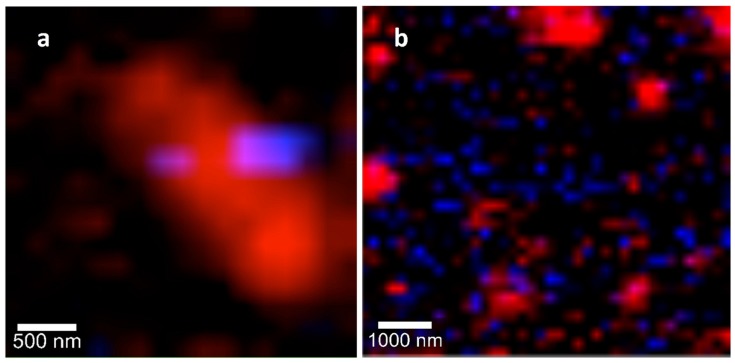
Confocal Raman spectroscopy images obtained by combination of stretching C-H bands from microorganism (red) and the Ag-N bands assigned to the PchNPs (blue): (**a**) *E. coli*, (**b**) *C. albicans*.

Then, interaction assays between microorganisms and silver nanoparticles were carried out using confocal Raman microscopy. Changes in cellular composition can be monitored using changes in Raman band profiles, which can be associated with the morphological changes of microbial cells, providing an image of the antimicrobial effect [24]. Recent reports have shown that Raman difference spectroscopy is able to provide molecular details on changes within *E. coli* cells caused by antibiotics, hydrogen peroxide [20,25] or graphene oxide [19]. In this work, the interaction of PchNPs with *E. coli* caused a decrease in the intensity of most Raman bands, while other remain the same (Table 1). The significant decrease in fatty acid Raman bands of *E. coli* cells could be associated to the loss of the cell membrane integrity after the PchNPs treatment, as observed in ESEM and TEM images (Figure 7 and Figure 8, respectively). In the case of the interaction with *C. albicans*, all bands of the Raman spectrum decreased in intensity. In addition, hyperspectral images were obtained in order to visualize the main characteristics of the interaction between the PchNPs and the microorganisms (see Figure 9). These images were obtained from the combination of the bands corresponding to the C-H stretching of the microorganisms and the bands corresponding to the Ag-N stretching of the PchNPs (Raman spectra are shown in Suplementary Materials, Figures S1 and S2). As shown in Figure 9a, the hyperspectral image shows PchNP interaction with an *E. coli* cell, providing structural information, which is comparable, and complementary to that obtained from the TEM and ESEM images.

### 2.7. Antibiofilm Activiy of PchNPs

The biofilm biomass of *E. coli* and *C. albicans* was measured with crystal violet stain. Comparison of the basal measures (biofilm without PchNP) with treated biofilm allowed analyzing the nanoparticles ability to eradicate biofilms.

**Figure 10:**
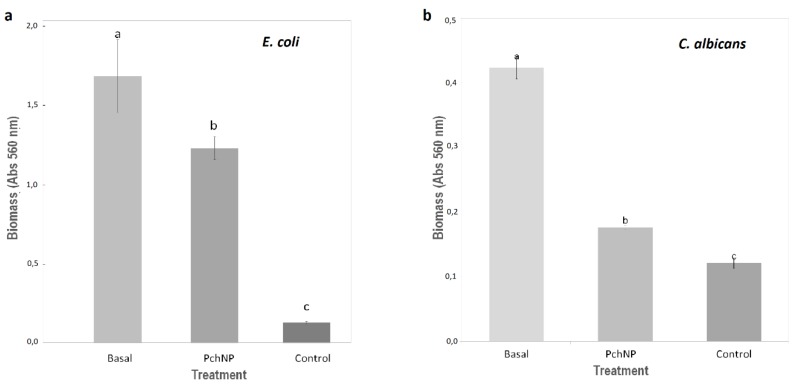
Activity of PchNPs against (**a**): *E. coli* and (**b**): *C. albicans* biofilms. Different letters represent significant differences at *p* < 0.05 probability level, according to ANOVA and Tukey’s test. *Basal*: biofilm without PchNPs, *PchNP*: biofilm treated with PchNPs, *Control*: No biofilm.

Reports on the antibiofilm activity of silver nanoparticles have been promising; showing that exposure to silver nanoparticles produced changes to the structural biofilm conformation of *Candida albicans* [27] and effectively inhibited *E. coli* and *Pseudomonas aeruginosa* biofilm formation [24]. Unfortunately, silver nanoparticles are prone to aggregation, reducing their antimicrobial efficacy and therefore surface functionalization before application is important to prevent aggregation and enhance their killing efficacy in biofilms, and reduce their cellular uptake and cytotoxicity. So natural and environmentally benign compounds, such as extracellular fungal compounds used in this work for the nanoparticle synthesis, could constitute effective stabilizing agents enhancing bacterial killing in biofilms and reducing cytotoxicity of silver nanoparticles [14]. According to the results, the biogenic nanoparticles synthesized in this work were able to eradicate the *E. coli* and *C. albicans* biofilms (Figure 10). Future works will include optimization of these biofilm eradication experiments, evaluating the effect of different treatment condition for biofilms, including incubation time and nanoparticle dose.

PchNPs produced 29% reduction in biomass of *E. coli* biofilm (Figure 10a) and 80% reduction in biomass of *C. albicans* biofilm (Figure 10b). *Candida albicans* virulence is a result of its capacity to form biofilms, a complex of cells, DNA, polymeric matrix, etc., on the surface of biomaterials and on catheters and prosthetic devices. The polymeric matrix has a crucial role in the antifungal resistance since it prevents the antimicrobial penetration inside the biofilm, allowing the internal cells to survive. Due of their intrinsic resistance to almost all antifungals in clinical use, increased resistance to this therapy, and the ability of cells within biofilms to withstand host immune defenses, the antifungal resistance of biofilms results most probably from the conjunction of several mechanisms that act in a time-dependent manner. Previous reports showed the antibiofilm activity of nanoparticles depends on their physicochemical properties, such as the size [28]. Similar to previously reported research by these authors, PchNPs of ca. 45 nm were able to eradicate biofilm of *C. albicans*. From these results, the biogenic silver nanoparticles synthesized in this work would be a useful tool for the development of biofilm disruption materials.

## 3. Materials and Methods

### 3.1. Synthesis of Silver Nanoparticles

Strain of *Phanerochaete chrysosporium* (12G) from the Cátedra de Microbiología General Collection CCMG, Facultad de Química (Montevideo, Uruguay), was used for the nanoparticle biosynthesis.

The mycelia were grown in Potato Dextrose Agar (PDA, BD Difco, Sparks, MD, USA) at 28 °C and two plugs of 0.9 cm in diameter were then transferred to 500 mL flasks containing 100 mL Potato Dextrose Broth (PDB, BD Difco). Fermentation was carried out at 28 °C, with agitation on an orbital shaker operating at 150 rpm for 72 h. The biomass from cultures was harvested by filtration and then washed extensively with sterilized distilled water to remove any remaining media components. Then, synthesis of silver nanoparticles was carried out as described in Sanguiñedo et al. [7]. Wet fungal mycelia were suspended in sterilized distilled water (0.1 g/mL) and incubated with agitation on an orbital shaker operating at 150 rpm. Then, the cell-free filtrate was collected by filtration of this suspension through membrane filter with 0.45 µm pore size. Finally, 50 mL of the cell-free filtrate was added to 50 mL of a silver nitrate solution. The mixture was incubated in dark. The absorbance spectrum was measured in the range of 250–800 nm and the maximum peak was determined, at different times. The reaction was stopped when there was no increase in the maximum absorption peak of silver nanoparticles. The remaining cell-free filtrate was used as control. Percentage yield was calculated according to weight of lyophilized silver nanoparticles and weight of silver nitrate used in the reaction, as previously reported [26].

To evaluate the incidence of the reaction variables in the biosynthesis of the nanoparticles, the following experimental conditions were modified: incubation time of the mycelia with water, concentration of AgNO_3_ and incubation temperature in the synthesis reaction.

After the synthesis reactions, the samples were centrifuged at 10,000 rpm for 10 min. The supernatant was removed and nanoparticles (PchNPs) were washed twice using sterilized distilled water, by centrifuging the nanoparticles for 10 min at 10,000 rpm. The absorbance peak of the purified silver nanoparticles was measured and the concentration was estimated according to Paramelle et al. [29].

### 3.2. Characterization of Silver Nanoparticles

#### 3.2.1. UV-Vis Spectroscopy

The absorbance spectrum was measured in the range of 250–800 nm, at predetermined time intervals. Also, the color changes of reaction mixtures were used as evidence for silver nanoparticles formation.

#### 3.2.2. ζ-Potential and DYNAMIC Light Scattering (DLS)

The measurement of ζ-potential and the hydrodynamic diameter of the nanoparticles were determined by Dynamic Light Scattering (DLS) utilizing a Zetasizer (Malvern Instruments, Malvern, UK). Samples were prepared at pH 6, in Milli-Q water. For ζ-potential determination, each sample was measured at 25 °C, three times, combining 10 runs per measurement. In the case of DLS, each sample was measured at 25 °C, 10 times, combining five runs per measurement. Results were treated using the Malvern Zetasizer software (Malvern, UK).

#### 3.2.3. Small Angle X-ray Scattering (SAXS)

An model Ultima IV X-ray powder diffractometer (Rigaku, Tokyo, Japan) using *CuK_α_* = 1.5418 Å radiation was used for the small angle X-ray scattering measurements. They were made at low angle in Bragg-Brentano geometry, applying an offset of 0.08^o^ in order to get the SAXS signal, on nanoparticle deposits on silicon substrate, with measurement ranges of *q* = 0.05 to 1.50 Å^−1^.

#### 3.2.4. Confocal Raman Microscopy

An aliquot of PchNPs was deposited on an aluminum support and dried at room temperature for a further analysis by confocal Raman microscopy. The measurements were made on an Alpha 300 RA WITec Raman microscope (WITec GmbH, Ulm, Germany) using a *λ* = 532 nm excitation laser wavelength and focused through a 100× objective.

#### 3.2.5. Colloidal Stability Assays

The colloidal stability of PchNPs was studied at different and ionic strength (10–500 mM NaCl) and pH (3–10) conditions by the measurement of the absorbance spectrum in the range of 200–800 nm.

### 3.3. Antimicrobial Activity of PchNPs

#### 3.3.1. Antibacterial Activity against *E. coli*

In order to determine the antibacterial activity of PchNPs, a bacterial inoculum (1 × 10^6^ CFU/mL) of *Escherichia coli ATCC 25922* in LB media was supplemented with different concentrations of PchNPs and a blank sample (bacteria without PchNPs) was also included in the assay as negative control, in a 96-well plate. Once the microbial cultures had been grown for a total of 24 h, 30 μL of 0.1 mg/mL resazurin (7-Hydroxy-3H-phenoxazin-3-one 10-oxide) in LB media was added to each well and incubated in the dark at 37 °C for 1 h under stirring.

##### Enviromental Scanning Electron Microscopy (ESEM)

*E. coli* cells were incubated with PchNPs (0.12 and 0.25 nM), in the same way as in the resazurin assay. Then, three wells of 200 µL each were mixed into an Eppendorf and centrifuged at 1400 rpm (300 G) for 10 min. The supernatant was removed and, for fixation of the cells, the pellet was resuspended into 1.5 mL of 2.5% glutaraldehyde in phosphate buffer 10 mM pH 7.2. The solutions were left for 2 h in the wheel. Then, the cells were washed once with 1.5 mL of sterile PBS and three times with sterile distilled water to remove glutaraldehyde. Finally, the pellets were resuspended in 200 µL of sterile MilliQ water. Data were collected on a Quanta FEG-250 (FEI Company, Hillsboro, OR, USA.) field emission SEM for high-resolution imaging working in ESEM mode using a GSED detector under high relative humidity conditions.

##### Transmission Electron Microscopy (TEM)

Samples were prepared as in the ESEM assay, including the PchNPs concentrations. The pellets were resuspended in sterile distilled water, 4 μL of the sample was deposited onto a carbon-coated copper grid (Cu200 mesh) and left to dry in air for several hours at room temperature. TEM analysis was carried out in a TECNAI T20 electron microscope (FEI) working at 60 kV.

#### 3.3.2. Antimicrobial Properties of PchNPs Using Confocal Raman Microscopy

Minimum inhibitory concentration (MIC) of the nanoparticles was determined by the microdilution technique according to the Clinical and Laboratory Standards Institute [30] in a 96-well plate (sterilized, 300 µL capacity, MicroWell, NUNC, Thermo-Fisher Scientific, Waltham, MA, USA), against the following microorganisms: *Escherichia coli ATCC 25922,* and *Candida albicans ATCC 101231.* The initial solutions of the nanoparticles were prepared in water and further serial dilutions were performed. The MIC was determined as the lowest concentration of silver nanoparticles that inhibited the visible growth of a microorganism after 24 h of incubation.

Studies of the phenotypic profile of microbial cells before and after treatment with silver nanoparticles were carried out using confocal Raman microscopy as previously described for antibiotics [28]. The cell suspensions were deposited on an aluminum support and dried at room temperature. The measurements were made using a 532 nm laser focused through a 100× objective. The data processing and statistical analysis was performed by principal component analysis (PCA) using a script of the research group. The Raman spectra (phenotypic profile) of the cells treated with the nanoparticles were compared to those of the untreated cells (control).

### 3.4. Antibiofilm Activiy of PchNPs

Assays against *E. coli* and *C. albicans* biofilms were carried out in a 96-well plate (sterilized, 300 µL capacity, MicroWell, NUNC), with six replicates.

A cell suspension of *E. coli ATCC 25922* (1 × 10^7^ cells/mL) was prepared and diluted to 1/10 in Nutrient Broth. Suspension was deposited in the 96-well plate and incubated 24 h at 37 °C. Control wells contained only Nutrient Broth. For *C. albicans* assay, plates were pretreated with 50% Fetal Bovine Serum (FBS) in Potato Dextrose Broth (PDB) at 37 °C for 30min and washed with 10mM PBS (pH 7.2). Then, a cell suspension of *C. albicans ATCC 101231* (1 × 10^6^ cells/mL) was prepared and diluted to 1/10 in culture broth. Suspension was deposited in the 96-well plate and incubated 24 h at 37 °C. Control wells were pre-treated with FBS and contained only PDB.

Then, supernatant was removed and the PchNPs solutions were added. Fresh culture broth was added to the basal biofilm wells. The plate was incubated 48 h at 37 °C. Supernatant was removed, the wells were washed with distilled water and dried at 50 °C for 40 min. Crystal Violet was added to each well and left at room temperature for 3 min. The contents of the wells were washed with water and resuspended in ethanol:acetone (70:30). The biofilm was quantified by measuring the absorbance at 560 nm and statistical analysis was carried out by Variance Analysis and Tukey Test.

## Figures and Tables

**Table 1:** Raman band assignment of microbial cells, control and treated cells with PchNPs. The band assignment was made based on previous work [26].

Microorganism	Raman Shift (cm^−1^)	Band Assignment	Effect on Treated Cells
***E. coli***	624	Nucleic acids	Diminishes
	810	Aminoacids	Diminishes
	982	Polysaccharides	No changes
	1186	Polysaccharides	Diminishes
	1243	Polysaccharides	Diminishes
	1308	Aminoacids or nucleic acids	Diminishes
	1344	Aminoacids or fatty acids	No changes
	1593	Fatty acids	Diminishes
***C. albicans***	670	Nucleic acids	Diminishes
	788	Aminoacids	Diminishes
	885	Aminoacids	Diminishes
	999	Polysaccharides	Diminishes
	1100	Polysaccharides	Diminishes
	1319	Aminoacids or nucleic acids	Diminishes
	1596	Fatty acids	Diminishes

## Supplementary Materials

The following are available online, Figure S1. Raman spectra for *E. coli* cells, control and treated cells with PchNPs, Figure S2. Raman spectra for *C. albicans* cells, control and treated cells with PchNPs.
