# Supplementary material for: Biofilm Eradication Using Biogenic Silver Nanoparticles
Source: Molecules. 2020 Apr 26;25(9):2023. doi: 10.3390/molecules25092023 (PMC7249070; doi:10.3390/molecules25092023)
Supplement: Supplementary file 1 [file molecules-25-02023-s001.pdf]

Suplementary Materials

Biofilm Eradication Using Biogenic Silver Nanoparticles

María Belén Estevez [1], Sofía Raffaelli [1], Scott G. Mitchell [2,3,*], Ricardo Faccio [4] and Silvana Alborés [1,*]

[1] Área de Microbiología, Departamento de Biociencias, Facultad de Química, Universidad de la República, 11800 Montevideo, Uruguay; bestevez@fq.edu.uy (M.B.E.); sraffaelli@fq.edu.uy (S.R.)

[2] Instituto de Ciencia de Materiales de Aragón (ICMA), Consejo Superior de Investigaciones Científicas (CSIC)-Universidad de Zaragoza, 50009 Zaragoza, Spain

[3] CIBER-BBN, Instituto de Salud Carlos III, 28029 Madrid, Spain

[4] Centro NanoMat & Grupo Física, Departamento de Experimentación y Teoría de la Estructura de la Materia y sus Aplicaciones (DETEMA), Facultad de Química, Universidad de la República, 11800 Montevideo, Uruguay; rfaccio@fq.edu.uy

* Correspondence: scott@unizar.es (S.G.M.); salbores@fq.edu.uy (S.A.)

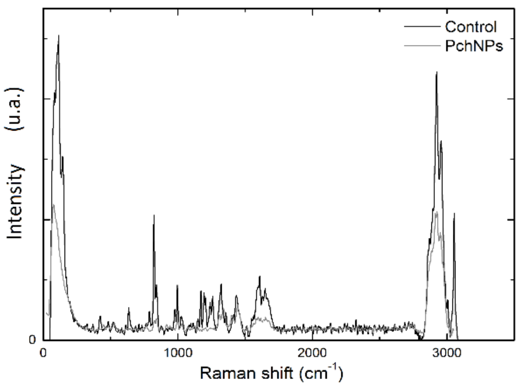

Figure S1. Raman spectra for *E. coli* cells, control and treated cells with PchNPs.

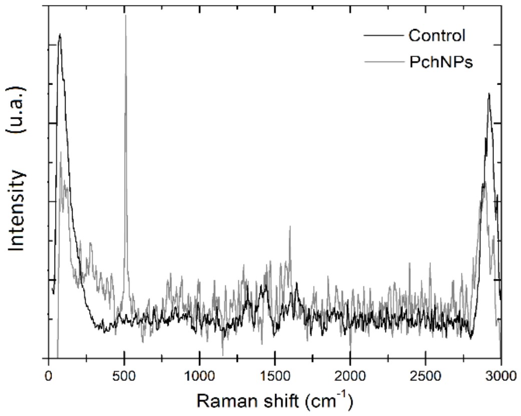

Figure S2. Raman spectra for *C. albicans* cells, control and treated cells with PchNPs.
